# Role of High-Fat Diet Alone on Lipids, Arterial Wall and Hippocampal Neural Cell Alterations in Animal Models and Their Implications for Humans

**DOI:** 10.3390/biology14080971

**Published:** 2025-08-01

**Authors:** Gayathri S. Prabhu, Mohandas Rao KG, Preethi Lavina Concessao, Kiranmai S. Rai

**Affiliations:** 1 Division of Anatomy, Department of Basic Medical Sciences, Manipal Academy of Higher Education, Manipal 576104, India; gayathri.bmv@manipal.edu; 2 Division of Physiology, Department of Basic Medical Sciences, Manipal Academy of Higher Education, Manipal 576104, India; preethi.concessao@manipal.edu (P.L.C.);

**Keywords:** high-fat diet, obesity, animal models, lipids, atherosclerosis, hippocampus

## Abstract

Background: A high-fat diet has been shown to have an impact on metabolism resulting in changes in arterial wall thickness and degeneration of surviving neural cells of the hippocampus. The present review focuses on the various animal models used to induce high-fat diet conditions for studying obesity-induced atherosclerosis, along with the associated changes observed in surviving neural cells of the hippocampus. It also highlights the limitations of rodent models and discusses their implications for human research. Methods: The sources for the literature search were Scopus, PubMed, Medline and Google Scholar. Both animal and human studies published were considered and are cited. Results: High-fat-diet-induced vascular changes, mainly in the tunica media, has been shown to have more impact on medium-sized arteries and on the Cornu Ammonis three subregions and outer dentatae gyrus of the hippocampus. Conclusions: High-fat-diet-induced neurovascular changes have been studied radically in animal models, and more supporting studies representing preclinical research should be advanced to humans.

## Simple Summary

High-fat-diet-induced obesity remains one of the major concerns globally, regardless of awareness. It directly impacts metabolism leading to changes in the lipid profiles with an increase in serum cholesterol, triglyceride and low-density lipoprotein levels affecting the structure of arterial walls. This results in atherosclerotic changes within the arterial wall. Moreover, the neurodegenerative changes, especially in 3 subregions of Cornu Ammonis and outer dentatae gyrus of the hippocampus, have shown to be directly associated with high-fat-diet intake. These neurovascular changes due to a high-fat diet have been extensively studied with animal models and in preclinical research. It is important to understand the detailed mechanisms involved in the metabolic changes due to a high-fat diet and to understand the various high-fat-diet models used to study these metabolic alterations. Additionally, the correlation between the atherosclerotic changes and neural cell degeneration is one of the important links that needs to be explored for detailed effects of lipid changes mainly due to high-fat-diet intake. Therefore, the present review thoroughly explains the metabolic changes implied for a better insight into high-fat-diet-induced neurovascular changes in both animal models and clinical research.

## 1. Introduction

Obesity has been one of the leading causes of cardiovascular and neurological deficits since childhood. Early childhood high-fat-diet (HFD)-induced obesity continues to have a negative effect on growing neural cells, as well as vascular structure, from an early stage. The present review focuses on the various diets used to study the causes and effects of HFD, and the adverse effects of HFD-induced obesity on arterial wall structure and hippocampal neurons from childhood to adulthood. It also discusses the relationship between HFD-induced arterial wall and hippocampal neural cell alterations in rodent models and their implications for humans.

### 1.1. Obesity Outcomes—Childhood to Adulthood

Obesity is a metabolic syndrome defined as an abnormal accumulation of fat in the body. One of the parameters used to assess obesity is body mass index, which is directly influenced by high-fat diets [1]. The essential cause of obesity is an imbalance between the consumption of fatty food and fewer calories expelled. Even though the awareness of the outcomes was present earlier, the severity of the problem is evident only now [2]. Studies have shown that the prevalence of overweight individuals will double among adults aged 20–69 years between 2010 and 2040. This will lead to a threefold increase in the number of obese individuals, including both men and women, by 2040. Interestingly, these studies have also reported that there is an increase in obesity among the rural population compared to that in urban areas [3]. Furthermore, childhood obesity is characterized by an increase in body mass index from an early age, which can have negative consequences on growth and development. This further continues in adulthood, and can lead to cardiovascular and neurovascular deficits. Additionally, lifestyle changes have raised concerns about increased intake of lipids that add to body fat. Total fat intake should be less than 40% of total energy, because the intake of HFD can increase disorders related to lipid breakdown, such as increased visceral fat, hyperlipidemia, and insulin resistance. High-fat-diet feeding can accelerate the increase in reactive oxygen species (ROS) through nicotinamide adenine dinucleotide phosphate (NADPH) oxidase activation. Increased ROS can cause oxidative damage to nucleic acids, proteins, and lipids. This further disrupts cellular homeostasis and leads to an increase in metabolic syndrome. Each of the effects mentioned will be discussed further.

### 1.2. Aim

The aim of the present review is to discuss the various animal models used to induce high-fat-diet conditions for studying obesity-induced atherosclerosis, along with the associated changes observed in surviving neural cells of the hippocampus. It also highlights the limitations of rodent models and discusses their implications for human research.

### 1.3. Research Question

Is there an association of high-fat-diet-induced neurovascular changes in animal (rat and mice) models with humans?

## 2. Materials and Methods

### 2.1. Keywords

High-fat diet, animal models, normal diet, fatty diets, lard, diet composition, dietary lipids, lipid parameters, arterial wall, tunica intima, tunica media, thickness of arterial wall, collagen and elastic fibers, arterial lumen, foam cells, macrophages, fatty streaks, atherosclerosis, hippocampus, CA1, CA3, dentate gyrus of hippocampus, behavioral changes, learning and memory, and obese humans were the key words used during the literature search.

### 2.2. Search Criteria

The sources for the literature search were Scopus, PubMed, Medline and Google Scholar. Both animal and human studies published were considered and are cited properly, according to their relevance for discussion. Articles written in English were considered for the review. Only articles appropriate for key words were considered. Articles were selected and limited based on the titles, abstracts, materials and methods pertaining to the key words only. A total of 200 articles were shortlisted, of which appropriate articles for the results of the present review were considered. Articles that were not written in English were not considered. Articles that were unpublished or only abstracts were not included in the study. The selected articles are summarized based on the animal models used, the experimental designs, the results and the discussion of the results in the articles.

## 3. Results

The results shown in Table 1, Table 2 and Table 3 summarize the animal models used to induce a high-fat diet, the animal models used to analyze the vascular effects of a high-fat diet, and the animal models used to analyze the neuronal cell changes in the hippocampus due to diet-induced obesity, respectively.

## 4. Discussion

### 4.1. High-Fat Diet in Animal Models

Studies have shown that rats fed with HFD for 12 weeks have increased serum levels of glucose and insulin, free fatty acids (FFAs), total cholesterol, HDL-C, LDL-C, and TG [4]. An imbalanced diet is the main factor that can target cardiovascular diseases. An increase in the incidence of cardiometabolic disease is observed, because HFD is currently preferred in terms of dietary habits. Even during pregnancy, high-fat dietary patterns can cause early childhood obesity in offspring [32,33]. The epigenetic changes in the liver and adipose tissue of the offspring due to maternal consumption of HFD during pregnancy indicate that maternal obesity can be genetically transferred, due to the transgenerational accretion of many epigenetic alterations, with histone methylation [34]. Diets rich in saturated fats are considered unhealthy or a fatty diet. Studies have shown that omnivorous species, including mice and humans, emphasize protein consumption more than carbohydrate and fat consumption [35]. Rodents fed with HFD are frequently used in research to exhibit human metabolic changes. Wistar/Sprague Dawley rats and C57BL/6 mice have been widely used as models of diet-induced obesity to study the mechanisms involved in the changes induced by HFD, as they are comparatively more susceptible to metabolic damage [36]. It is reported that fatty acids use more than half of the calories from fat made up of lard and beef tallow which are rich in saturated fatty acids. In rodent studies, when these diets are used, the energy concentration is greater than that of normal control diets, which results in obesity [37].

Male mice are most used in experiments because they are more susceptible to HFD-induced insulin resistance than are females. When an HFD is given for a particular period, cellular dysfunction at the level of muscles and liver is observed. These effects may include hyperinsulinemia, increased triglyceride levels, beta-cell dysfunction, muscle hypertrophy, and metabolic changes in the liver. Therefore, the dietary induction of obesity involves both adipocyte hyperplasia and hypertrophy. However, the other feature of obesity starts to appear after approximately 4 weeks of HFD exposure [5].

In contrast, studies have shown that HFD in C57BL/6J mice results in low grade inflammation and stenosis. This also results in additional caloric consumption, hepatic steatosis, lipid increase in muscle, pancreas, and heart tissue and impaired neurological function. These findings represent some of the major diseases observed in aging humans. This alone shows that overconsumption of calories alone is a major risk factor for numerous diseases. There are reports that pure HFD does not lead to nonalcoholic steatohepatitis (NASH) in mice. However, HFD, along with increased cholesterol and fructose in the diet, is required for hepatocellular changes and fibrosis [6].

It is postulated that when HFD is given to Sprague Dawley male rats during the postnatal period after 21 days of weaning for a duration of 3 months, the body mass index is significantly greater than that of rats fed with a normal chow diet. This indicates that an increase in the body mass index is an important parameter in the early detection of obesity [7]. In addition, research has shown that severe leptin resistance is a major factor in the development of chronic hyperglycemia, which can lead to obesity and insulin resistance in leptin receptor-deficient Zucker rats, in addition to hyperglycemia [8]. Similar effects of high-fat-diet supplementation showing an increase in total cholesterol, LDL, and TG levels have been reported in studies conducted on pig models [38]. However, in this review only rat/mice models were considered, and further reviews can be carried out on various animal HFD models for detailed elucidation of the mechanisms.

### 4.2. High-Fat Diet and Changes in Arterial Wall Structure

One of the main causes of cardiovascular risk due to HFD is a change in the arterial wall structure, which can lead to hypertension. Many types of animal models are available to distinguish between obesity due to HFD consumption and hypertension. The ability of rat and mouse models to induce diet-induced obesity after the weaning period has also varied. The Dahl salt-sensitive (SS) rat has been associated with hypertension due to adiposity. This study confirmed that high-fat feeding in males after weaning produced hypertension. Female Dahl SS rats also developed similar hypertension when fed the same fat diet beginning at weaning [9]. Furthermore, studies have shown that diet-induced obesity shows a common association between inflammation and endothelial cell damage in coronary arteries. Metabolically unhealthy individuals showed a greater risk for endothelial cell damage than did metabolically healthy people [39].

### 4.3. Basic Structure of Medium-Sized Arteries

**Figure 1:**
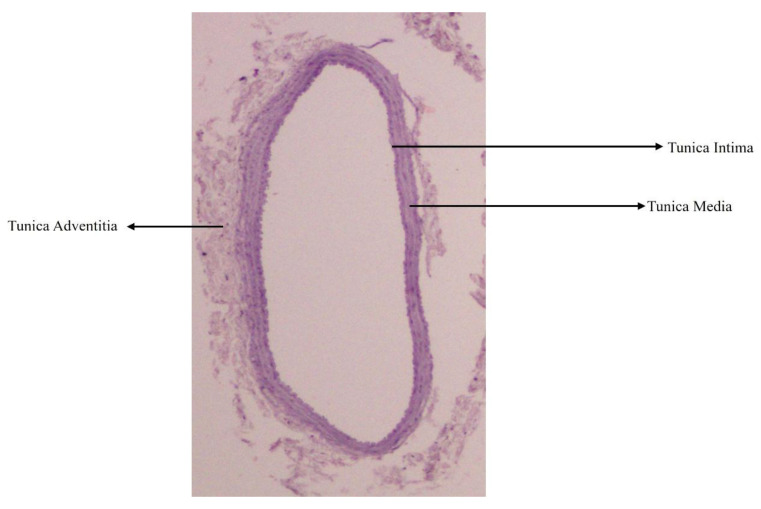
Representative photomicrograph of transverse section of a medium-sized artery showing tunica intima, media, and adventitia using Haematoxylin and Eosin stain from an 8-month-old Sprague Dawley rat (40×) [40]. (Reproduced from Gayathri et al.)

**Figure 2:**
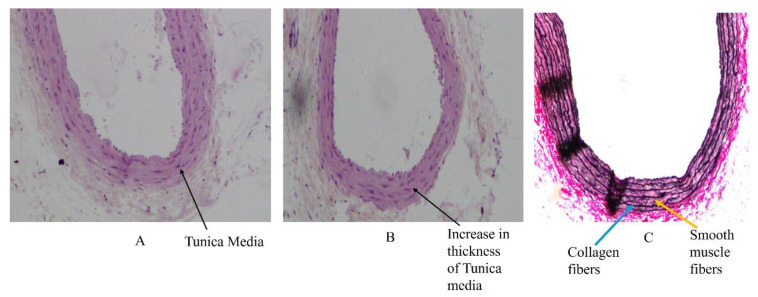
(**A**) Representative photomicrograph of transverse section of medium-sized artery representing tunica media using Haematoxylin and Eosin stain from an 8-month-old Sprague Dawley rat (40×) [40]. (Reproduced from Gayathri et al.) (**B**) Representative photomicrograph of transverse section of medium-sized artery representing increase in thickness of tunica media using Haematoxylin and Eosin stain from an 8-month-old Sprague Dawley rat (40×) [40]. (Reproduced from Gayathri et al.) (**C**) Representative photomicrograph of transverse section of medium sized artery representing changes in arrangement of smooth muscle fibres and collagen fibres in tunica media using Verhoff-Vangeison stain from an 8-month-old Sprague Dawley rat (40×) [40]. (Reproduced from Gayathri et al.).

Structurally, an arterial wall is made up of three layers, namely tunica intima, consisting of endothelial cells supported by loose connective tissue, tunica media, made up mainly of circular layers of smooth muscle cells, elastic and reticular fibres, and tunica adventitia, consisting of collagen and elastic fibres. The tunica media starts to show metabolic changes when serum lipids are altered leading to increase in its thickness (Figure 1) [40]. These layers are well understood, through a transverse section of an artery using Haematoxylin and Eosin, and Verhoff and Vangeison stains (Figure 2A–C) [40].

### 4.4. Primary Changes in the Tunica Intima and Tunica Media of Arteries Due to High-Fat Diet

When animals fed with HFD become obese, diet-induced changes such as vasoconstriction and hyperleptinemia can be observed. Nitric oxide production occurs through increased activation by leptin, which is partially mediated by the Akt pathway [10]. Atherosclerosis (a type of arteriosclerosis) is a silent progressive process of cardiovascular disease, and is associated with large and medium-sized arteries. An increase in the accumulation of cholesterol and TG in the intimal walls of arteries leads to a reduction in arterial lumen size. To determine the atherosclerotic effects on the arterial wall, studies have been conducted using different models of HFD in animals. DYETs and local foods were used to induce obesity in male rats, which increased BMI, lipid profile and vascular wall thickness in them [11]. Studies have shown that changes in the arterial wall that are initiated due to atherosclerosis are directly related to the impact of diet consumed. Areas in which lipoprotein accumulation is high have a high mechanical force, which increases the residence time of moving atherogenic particles at the lumen surface. An increase in the accumulation of LDL is observed before macrophage foam cell formation, which leads to increased tunica intima thickness. With increasing endothelium thickness, smooth muscle proliferates toward the tunica media. Many of the morphological changes are a result of subendothelial collection of macrophage foam cells and lipids. Smooth muscle cells accumulate during the initial stage of lesion formation and are integral to changes in smooth muscle structure. Within lesions, macrophages activate inflammatory responses that involve lipid proteins, which are characteristic of oxidized lipoproteins [41].

**Figure 3:**
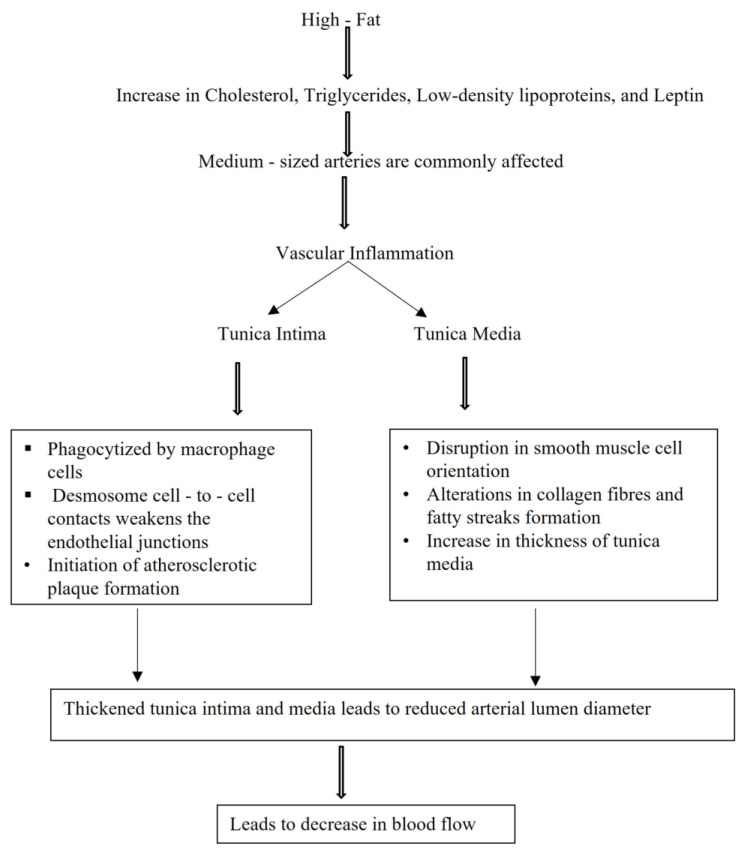
Flow chart describing the sequence of processes involved in atherosclerosis resulting from the intake of a high-fat diet.

The above-mentioned atherosclerotic effects are summarized in Figure 3.

Studies have shown that HFD containing lard induces alterations in the tunica intima and tunica media of the common carotid artery (CCA). This is because the CCA is a medium sized artery and is commonly involved in atherosclerotic changes. The hypertrophy of smooth muscle cells in the tunica media, along with the scattering of collagen and elastic fibers, results in an increase in the thickness of the tunica media. Subsequently, along with fatty steaks, the foam cells push toward the tunica intima, leading to a decrease in luminal diameter. Collagen fibers can increase during atherosclerotic plaque formation, further increasing the thickness of the tunica media. These early effects after the weaning period have shown acute and alarming effects of dietary changes on the thickness of the arterial wall. Furthermore, carotid artery plaque formation was associated with visceral fat and obesity. The atherosclerotic plaque formation was not always associated with body mass index [42].

In addition to the information on arterial wall structure in diet-induced obesity, studies have reported large artery lesions and cardiac fibrosis. This effect in experimental animals has been observed to be like that in humans. Male Wistar rats fed with HFD for 10 weeks showed pathological changes, such as cardiac muscle relapse with fat deposition, mononuclear cell infiltration, fat deposition in the arterial wall, and cardiac muscle degeneration with inflammatory cell infiltration. These effects from an early-age period must be identified to further reduce the detrimental effects on arterial wall and heart structure [12]. Experiments were also conducted using HFD in animals in which dietary intervention for 12 weeks also resulted in heart fibrosis. In the postweaning period, HFD-induced obesity alters cardiac structure, which is intervened by oxidative stress, inflammation, apoptosis, and the TGF-β1/Smads signaling pathway, as well as by an increase in ox-LDL correlated with atherogenesis [13]. HFD-induced obesity beginning in childhood causes adverse effects on BMI and lipid profiles, further leading to arterial wall changes and early atherosclerotic lesion formation.

### 4.5. Oxidative Stress on Arterial Wall, Endothelial Dysfunction and Hippocampal Neuronal Cell Alterations

Oxidative stress is an imbalance between reactive oxygen species and the antioxidant defense system. It plays a significant role in endothelial dysfunction and alterations in hippocampal neurons. Increase in oxidative stress is due to increased production of reactive oxygen species and LDL oxidation. This leads to atherosclerotic formation, leading to inflammation in the arterial wall. Each of these are linked to reduced bioavailability of nitric oxide, which alters the gut through mitochondrial dysfunction, along with altered proteostasis [43]. Additionally, alterations in reactive nitrogen species production and antioxidant defense mechanisms also play a key role in vascular changes, especially in the brain. This is due to the inefficient electron transport chain in mitochondria, leading to significant reactive oxygen species production. Non-replication of neuronal cells and their low repair capacity increase susceptibility to oxidative stress. This influences cerebral vascular tone and permeability [44]. Endothelial dysfunction leads to infiltration of LDL into the subendothelial layer, leading to its accumulation and subsequent oxidation to ox-LDL. This activates the expression of cell adhesion molecules like VCAM1 and ICAM1. These molecules move towards inflammatory immune cells and into the sub-endothelial space, leading to chronic inflammation [45]. Furthermore, it is observed in laboratory animals that mitochondrial dysfunction, oxidative stress and cardiac hypertrophy are related to a high-fat and sucrose diet. This increased oxidative stress induced by HFD has been observed with hydrogen peroxide formation, resulting in endothelial dysfunction, wherein nitric oxide production was suppressed and disrupted [46].

### 4.6. Influence of High-Fat-Diet-Induced Arterial Wall Changes on Neural Cells in Hippocampal Subregions

**Figure 4:**
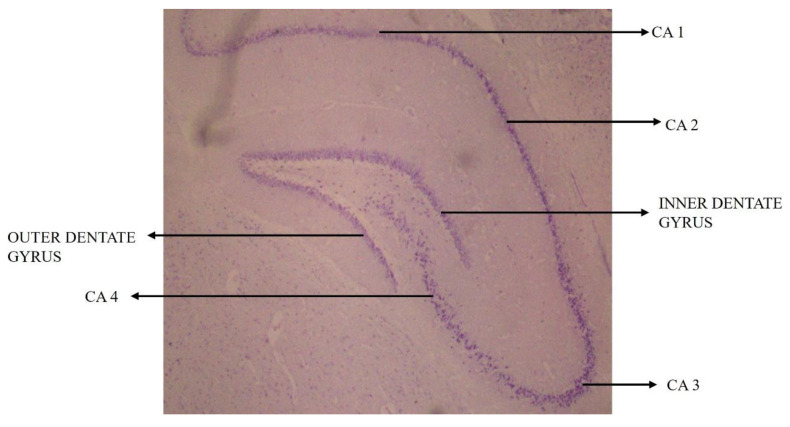
Representative photomicrograph of sub-regions of hippocampus of rat brain from an 8-month-old Sprague Dawley rat (40×) [40]. (Reproduced from Gayathri et al.)

**Figure 5:**
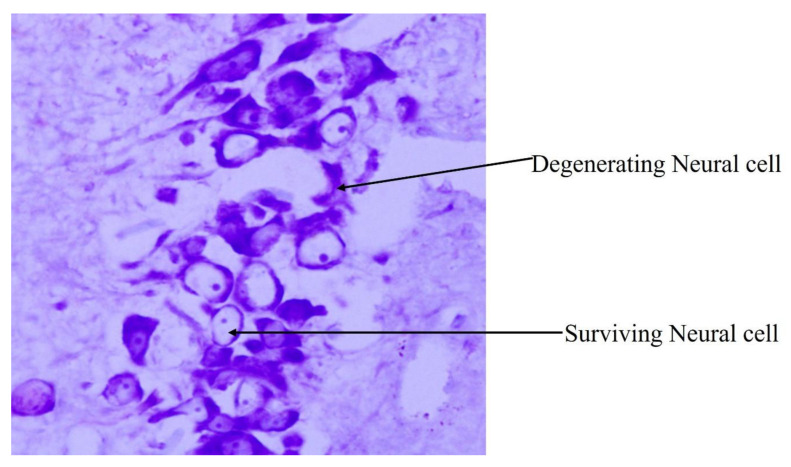
Representative photomicrograph of surviving and degenerating neural cells in CA3 subregion of hippocampus of rat brain from an 8-month-old Sprague Dawley rat (40×) [40]. (Reproduced from Gayathri et al.)

The CA (Cornu Ammonis) of the hippocampus proper consists of CA1, CA2, CA3 and CA4, regions along with inner and outer dentatae gyrus (Figure 4) [40]. When a section of rat brain is stained with Cresyl Violet, the normal neural cells usually appear to have a centrally placed nucleus and appear rounded to oval. However, degenerating neural cells show the flame-shaped, pyknotic cell body of pyramidal neurons. The flame-shaped cells, which look deeply basophilic, are indicative of karyopyknosis of the neurons of the hippocampus (Figure 5) [40].

Studies have shown that feeding with HFD for 16 continuous weeks in an ApoE−/− mouse model results in persistent atherosclerotic changes. This may be due to changes in the lipid metabolism pathway in the prefrontal cortex and hippocampus region, along with changes in the gut microbiota. Additionally, this study revealed the existence of a microbiota–gut–brain axis, and confirmed that changes in lipid metabolism pathways in the hippocampus were closely related to the gut microbiota in atherosclerosis [14]. In obesity, the gut microbia composition is altered, and neuroinflammation events are observed in reward-associated brain regions [47]. Increased permeability of the gut and blood–brain barrier induced by microbiota dysbiosis may mediate neurodegenerative disorders and cognitive impairment. Variations in the gut microbiota composition leads to peripheral accumulation of phenylalanine and isoleucine, which stimulates the differentiation and proliferation of proinflammatory T helper 1 cells and is associated with M1 microglia activation. This can be one of the reasons for neuroinflammation [48]. Additionally, epigenetic changes due to a high-cholesterol diet involve processes like DNA methylation, histone modifications though acetylation, methylation and noncoding RNA (ncRNA)-associated gene expression reduction. HFD also causes epigenetic dysregulation that influences gene transcription. An extended high-fat-diet intake induces gradual and fat-depot-specific DNA methylation changes in white adipose tissue, altered signaling and inflammation. This also results in increased production of ketone bodies from excess fat, which influence gene expression by modifying histones through histone post-translational modification. This is one of the key mechanisms linking diet to epigenetic changes [49].

Furthermore, in a study, 12-week-old male adult C57BL/6 J mice were fed either an HFD or a control diet for 7 weeks, and the effects of a HFD were shown to occur at the microvascular edge. This further resulted in a substantial depletion of pericytes, along with deceased exchanges between micro-vessels and perivascular microglia. This is also related to a lack of angiogenic response. One of the important highlights of HFD consumption is the direct involvement of neurodegenerative changes due to vascular dysfunctions [15]. In a study conducted using 8-week-old male and female C57BL/6J mice, a high-fat and high-sucrose diet, consisting of 60% fat and 20% sucrose through drinking water, from 10 weeks of age, was given for 6 months. The diet was reversed for 4 months, followed by controlled diet feeding for 2 months. This study revealed systematic modifications in cortical and hippocampal metabolites in HFD-fed mice. These changes indicated alterations in energy breakdown, alterations to counteract osmolarity changes, and mitochondrial stress, along with cortical neurodegeneration. It was reported that these variations followed within weeks after the onset of diet changes, an almost entire exchange of the studied phenotype after restoration to a healthier diet. Therefore, alterations in brain organization explain plasticity, which is altered relative to the permanent structural damage to brain tissue [16].

### 4.7. High-Fat Diet and Hippocampal Dysfunctions

HFD-induced obesity causes hippocampal dysfunction, leading to cognitive impairment. Hippocampal impairment was observed in six-week-old male Swiss mice fed with a short-term HFD. This is because of neuroinflammation and blood–brain barrier dysfunction, which could be due to the inhibition of the TNF-a inflammatory pathway, which mitigates behavioral changes [17]. Studies involving different durations of HFD consumption have also shown changes in the hippocampus. Early changes in brain integrity were detected in male Wistar rats fed either a normal diet or an HFD for 2, 8, 12, 20, or 40 weeks. At the end of the 12-week period, hippocampal synaptic dysplasticity and a decrease in dendritic spine density were observed. An increase in hippocampal reactive oxygen species with cognitive decline has also been reported. Furthermore, it has been reported that gut dysbiosis develops in the earliest phase of HFD consumption, followed by brain pathology. This further leads to cognitive decline in obese insulin-resistant rats [18]. A study using hIAPP transgenic mice fed HFD for 6 or 12 months showed increased amylin accumulation in the hippocampus, along with neural degeneration, brain aging, Aβ42 deposition, and impaired glucose consumption and cognition [19].

### 4.8. High-Fat-Diet Feeding and CA1 and CA3 Subregions of the Hippocampus

It has been reported that 21-day postnatal male Sprague Dawley rats fed HFD for 90 days showed a significant reduction in the number of surviving neural cells in the CA1, CA3 and outer dentate gyrus of the hippocampus compared to the same-age-matched normal control rats. This is because of the increased levels of serum triglycerides, cholesterol, and LDL, along with a reduction in decreased HDL, leading to an increase in adipocytes. Sterols, which are cholesterol derivatives, can cross the blood-brain barrier and boost the production of superoxide and free radicals when exposed to a high-cholesterol diet. This specifically impacts the hippocampal CA1 and CA3 subregions, which may further result in memory loss and decreased hippocampal neurogenesis [20].

C57BL/6 mice fed HFD for a period of 31 weeks showed a twofold increase in hippocampal oxidative changes. An increase in free radical formation and oxidative stress was also observed. Along with neuronal cell damage, alterations in the synthesis of apolipoprotein E have been reported. In addition, HFD has adverse effects on synaptic transmission, learning and memory, BDNF, and cognitive performance. This is caused by fatty diets leading to oxidative damage [21]. Studies have also reported that when male Sprague Dawley rats were exposed to HFD for an acute period of 7 days, they exhibited hippocampal damage, triggering memory impairments [22].

Swiss mice fed HFD for 1, 2 or 4 weeks showed alterations in hippocampal structure. An increase in proinflammatory cytokines and the permeability of the blood–brain barrier was observed after 2 days of supplementation with HFD. At 3, 5, and 7 days of dietary mediation, memory and learning impairments, changes in behavior, and alterations in synapses were observed. Furthermore, hippocampal alterations (after 4 weeks) included mitochondrial dysfunction and astrocytic activation. These results highlight the deleterious effects of HFD consumption on early damage to hippocampal neural cells. It has also been reported that mice that are continually subjected to lard-based HFD exploit glucose intolerance and decreased insulin sensitivity. Hippocampus-dependent spatial memory was disrupted by neurochemical alterations. Alternately, when compared with control mice, mice fed a 60%-fat but not a 45%-fat diet exhibited memory dysfunction in both HFD groups. Furthermore, the levels of proteins needed for sufficient synaptic function were found to be altered when HFD was induced. This suggests that synaptic alterations may occur in the brain when a high-fat diet is induced, without major changes in metabolite concentrations [23].

Similar findings were reported in research with 6-month-old female Fischer 344 rats that were fed HFD. The animals had decreased integrity of the blood-brain barrier (BBB) and increased microgliosis in the hippocampus. Occludin protein expression was found to be elevated in the mossy fibers of the hippocampal CA3 region, as well as in the dentate gyrus hilar neurons, and to be decreased in blood vessels. These deficits are mainly observed due to an increase in cholesterol levels and inflammation due to HFD [24]. Long-term exposure to HFD induced dyslipidemia, as well as decreased movement in middle-aged 37-week-old male Wistar-Kyoto rats. Moreover, chronic exposure to a fatty diet results in a reduction in the number of astrocytes, which leads to reduced brain resistance. Interestingly, in this study, the number of hippocampal pyramidal neurons remained unaffected [25]. In a study where male C57BL/6 mice were fed with HFD at 4 weeks of age and continued for a long period, deeply stained nuclei were observed in the CA1 and CA3 regions of the hippocampus, due to oxidative damage. A reduction in adiponectin (APN) and Nrf2 expression in the hippocampus was also observed, which is related to irregular oxidative stress and anxiety-linked behavior [26]. This can be due to increased oxidative stress, which can serve as one of the signals that trigger the NLRP3 inflammasome activation [50]. Nrf2 in its transcriptionally active form binds to the antioxidant response-element sequence present in the promoters of genes encoding enzymes involved in antioxidant defense and detoxification of various xenobiotics. This activation is important for macrophage survival, which can be disturbed if the expression occurs [51]. Expression of APN is reduced due to neuronal apoptosis leading to cognitive impairment [52].

Studies have shown that middle-aged male Sprague Dawley rats fed HFD are more susceptible to hippocampal dysfunction. Middle age is a specification of early brain aging for mitochondrial function, with the hippocampus being more prone to metabolic damage than the frontal cortex [27]. Some studies have also shown that the density of the molecular effect of the HFD on the brain hippocampal microvascular endothelium continues through the regulation of the expression of protein-coding and noncoding genes [28]. Chronic stress and HFD increase glucocorticoid production and affect CA3 apical dendritic arborization and synaptic activity. It also contributes to cognitive deterioration, due to hippocampal dysfunction resulting from decreased hippocampal dendritic spine density, fewer CA1 synapses, and reduced BDNF levels [29].

It is found that obesity involves genetic changes involving hypothalamic Iroquois homeobox 3 (Irx3) gene. This is altered by diet-induced obesity, wherein the levels of IRX3 are less than normal. This can influence the phenotype, due to fat intake, which, along with less energy expenditure, intensifies the obese phenotype due to a higher caloric intake and reduced energy expenditure [53]. HFD-induced obesity is linked to metabolic dysfunctions, and macrophage penetration in adipose tissue contributes to neuroinflammation and neuronal death. Dyslipidemia, insulin resistance, and hepatic steatosis can also intensify seizure-induced brain damage [54].

According to the above literature on the effect of an HFD, mainly on the thickness of the tunica media of the arterial wall and CA3 subregion of the hippocampus, studies have also reported a correlation between the two parameters and metabolic disturbances. Twenty-one-day-old male Wistar rats fed with HFD for a longer period exhibited hyperlipidemia and an increase in the thickness of the tunica media of the common carotid artery, indicating a positive relationship between the two parameters. This is because long-term induction of HFD alternates serum lipid levels, which directly initiates atherosclerotic changes resulting in lesion formation, thus resulting in atherosclerosis. As age increases, the stiffness of the arterial wall increases, resulting in arterial wall damage due to HFD. Furthermore, when HFD was given for a longer period, the tunica media showed relatively more damage. Therefore, HFD supplementation from a young age through adulthood linearly increases lipid profile levels and thickening of the tunica media of the arterial wall in adulthood, resulting in cardiovascular risks. Increase in TG, cholesterol and LDL levels resulted in significant reductions in surviving neural cells in the CA3 subregion of the hippocampus. This indicates a direct correlation between hyperlipidemia and surviving neural cells in the hippocampus [30]. Alternately, in a study where which male Wistar rats were fed with HFD for a period of 5 weeks, effects on the cholinergic system and cognitive impairments were observed. This is due to increased levels of choline acetyltransferase (ChAT) and vesicular acetylcholine transporter (VAChT) in obese rats. Furthermore, the acetylcholinesterase (AChE) enzyme is downregulated in the hippocampus [31]. There are studies exploring the effects of HFD in older age in which impaired spatial learning and memory and working memory are associated with decreased neurogenesis [55].

### 4.9. Correlation Between High-Fat-Diet-Induced Arterial Wall and Hippocampal Neural Cell Changes

Hippocampal vasculature and structure are vital in maintaining neurocognitive health. This is disturbed in metabolic disorders, leading to vascular cognitive impairment. While understanding the blood supply to the brain, especially the hippocampus, it is essential to note that there is less dense vascularization observed in the hippocampus. It is comprised of a series of consistently spaced varying arcs of arterioles and venules. This pattern could influence neuronal functioning, memory retention power and cognition. In addition, the decrease in lumen diameter can significantly increase vascular resistance and reduce perfusion to the nervous tissue, resulting in metabolic changes [56]. Additionally, it is reported that HFD and hypertension can generate neuroinflammation, oxidative stress, and angiogenic signals within the cerebral small vessels and adjoining cellular environment. These findings are important to understand the variation in metabolic factors in maintaining cerebrovascular health [57].

Hence, it is important to understand the connection between arterial wall changes, leading to a decrease in blood flow and causing neural changes in the brain. This becomes more eminent in arteries like the CCA, which is directly involved in blood supply to the brain and is more prone to atherosclerotic changes, as it is a medium-sized artery. As these changes bring in noticeable effects at the molecular level, the present review provides an insight for outlying experiments to explore and elucidate the mechanisms underlying HFD-induced changes in the arterial wall and neuroinflammation. Further, it confirms the firm correlation between high-fat-diet-induced structural changes in the arterial wall and the hippocampal neurons.

### 4.10. Limitations of High-Fat Diet in Animal Models for Human Studies

The most preferred diet for rodents was a lard-based HFD, and diets rich in saturated fatty acids and high levels of polyunsaturated fats also caused structural damage and cognitive deficits. This mainly affects hippocampus-dependent learning and memory, leading to cognitive impairment. In human epidemiological studies, a correlation between the consumption of HFD and cognitive impairment was observed, leading to neurodegenerative diseases. Interestingly, sex differences were observed in both animal and human studies, which were species dependent. In animal models, males are more prone to insulin resistance than females. Therefore, while comparing the mechanisms of HFD consumption pertaining to pathological changes, sex differences in mammalian species should not be directly generalized to humans [58].

It has been reported that various diets rich in fatty acids induce intestinal inflammation and permeability, due to dysbiosis of the gut microbiota. This can further lead to neural inflammation. In addition, dietary lipids increase perivascular adipose tissue, which causes malfunction of endothelial cells within the vessel wall, thereby increasing the risk of cerebrovascular disease. Therefore, different fatty acids can have a direct influence on the brain, and consuming diets low in meat and fibers can prevent diet-related noncommunicable diseases and conditions [59].

Furthermore, it has been reported that individuals exposed to both 45%- and 60%-fat diets develop neural changes resulting in a decline in neuro and glio transmission in the hippocampus. This further leads to impaired spatial memory performance. Neurochemical alterations are a result of distinct metabolic syndromes, due to variations in the percentage composition of lard [23].

Among the many animal models studied, mouse models have been shown to have better underlying genetic, epigenetic and environmentally induced mechanisms. This can lead to disease development and progression. The Ldlr knockout model resembles the human lipoprotein profile, and is therefore a suitable model for studying cholesterol and lipoprotein metabolism. Although atherosclerotic changes like those in animal models have been shown in humans, the progression of lesions to atherosclerotic rupture has not yet been observed [60].

In HFD-induced models, mechanisms of action are streamed within the diet involved or through gene expression due to the diet. Possible models for the later stages of plaque rupture and atherothrombosis have still not been reported in rodents. However, these animal models help in inducing human-like disease in a short span. Also, studies involving longer durations in animal models are challenging because of the cost of the experiment and differences in the mechanisms and metabolism involved in the anatomical and physiological basis of the disease. To understand the detailed pathophysiology, parallel research on humans and experimental animals can be carried out to obtain a correct idea of the pathophysiology of disease and reduce the gap between preclinical studies and human medicine [61].

Furthermore, animal models used to induce obesity are more beneficial, since various mechanisms of obesity can be studied in the preclinical phase. However, there are still limitations and applicability in humans with respect to physiological changes [62].

Many studies on diet-induced obesity in rats and mice have been conducted. However, the use of standardized protocols is lacking in these studies. The results also varied due to the time of intervention and the animal strains studied. However, it has been reported that diets with high amounts of saturated fatty acids show pathophysiological changes like those in humans [63]. This observation is also made in animal models used in diabetic research, wherein conventional diabetic animal models are not able to recapitulate similar pathophysiology and molecular mechanisms of disease [64]. Therefore, supporting the need for preclinical research using animals, animal models used in vascular research should be refined [65].

### 4.11. Present Prevention and Treatment Insights for Increased Lipid Profiles, Arterial Wall and Hippocampal Neuronal Cell Alterations

Metabolic syndrome due to impaired glucose metabolisms, unhealthy lifestyle and dietary intake can be maintained by targeting astrocytes to increase glutamate re-uptake and reduce the glutamate excitotoxicity. Reducing systemic inflammation due to increased lipid production can be controlled by maintaining the secretion of inflammatory cytokines and toxic byproducts [66]. A study has shown that combined supplementation of choline and DHA and exposure to enriched environment from an early young age significantly attenuates the negative impact of a high-fat diet on body mass index, serum lipids and thickness of the arterial wall [30]. Further, oral supplementation of essential brain nutrients has a more beneficial impact on the hippocampal neural cells, as well as on learning and memory [20].

## 5. Conclusions

The adverse effects of HFD remain persistent, even if HFD supplementation is stopped or continued for specific time frames. This indicates that the effects of HFD on vascular changes, mainly in the tunica media, remain resilient. HFD also caused hippocampal neural cell damage in the CA3 region and outer dentatae gyrus, leading to cognitive deficits in learning and memory. There is a strong correlation between high-fat-diet-induced structural changes in the arterial wall and the hippocampal neurons.

## Figures and Tables

**Table 1:** Summary data describing animal models for inducing high-fat diet.

Author	Year of Publication	Animal Model	Key Result
Zeynep Tuzcu [4]	2017	Wistar Rats	HFD feeding increased body weight, visceral fat, and liver weight and reduced feed consumption, as compared to the control rats
Pettersson [5]	2012	C57Bl/6 Mice	HFD-induced metabolic changes were seen less in female mice HFD-fed male mice showed an increase in adipose tissue inflammation, glucose intolerance, and an increase in insulin levels and hypertrophy of islet cells
Burchfield JG [6]	2018	C57BL/6J Mice	High-fat/high-sucrose diet (HFHSD) mice showed metabolic changes, hyperleptinemia, reduced physical activity, glucose intolerance, peripheral insulin resistance, hyperglycemia, increase in lipid deposition, and bone weakening
Prabhu. S [7]	2022	Wistar Rats	HFD when given for 3 months showed a significant raise in body mass index as compared to normal control same-age matched rats
M.A. Guzzardi [8]	2022	fa/fa Zucker Rats	Leptin resistance led to glucose intolerance and an increase in hepatic glucose production. This also led to changes in pancreatic and intestinal hormones. Thus, fat deposits in adipocytes and hepatocytes were also seen

**Table 2:** Summary data used for describing the animal models used to analyze the vascular effects of a high-fat diet.

Author	Year of Publication	Animal Model	Key Result
S.W. Watts [9]	2021	DahlSS Rats	HFD, when given as a diet from weaning period, led to development of hypertension in both male and female DahlSS rats. Endothelial dysfunction, along with medial hypertrophy, was observed.
Rocha [10]	2019	Wistar Rats	High-unsaturated-fat diet showed vascular sensitivity to leptin and increasing Nitric oxide bioavailability. This leads to increase in Nitric oxide production through an increase in NOS activation, which is partly intervened with by the Akt pathway.
Putro [11]	2021	Wistar Rats	Supplementation of DYETs that contained 40% of calories as a fat, lard and cholesterol diet, showed a higher increased internal carotid-artery vascular wall thickness.
Basma S. Ismail [12]	2022	Wistar Rats	HFD-administered rats showed fat vacuoles in intracytoplasmic regions and cardiomyocytes, mononuclear cellular infiltration, and arterial wall fat deposition with degeneration. Central necrosis and cardiac muscle disintegration were also observed, along with loss of striations in myocytes leading to fragmented cardiomyocytes.
A. Feriani [13]	2021	Wister Rats	Subchronic postweaning exposure to HFD or permethrin (PER) exposure can change cardiac integrity and initiate fibrosis. In addition, also, raised aortic levels of ox-LDL are seen.

**Table 3:** Summary data used for describing animal models used to analyze neural cell changes in the hippocampus due to diet-induced obesity.

Author	Year of Publication	Animal Model	Result
Hu et al. [14]	2022	ApoE−/− mice	The HFB group displayed symptoms of depression and clinical markers associated with atherosclerosis.
Elabi [15]	2021	C57BL6 mice	HFD in mice resulted in a significant depletion of pericytes.
Garcia-Serrano AM [16]	2022	C57BL/6J mice	Mice fed HFHSD, both male and female, exhibited elevated anxiety-like behavior, memory impairment in object identification tasks, but intact working spatial memory.
de Paula [17]	2021	Swiss mice	HFD resulted in depressive-like behavior or astrocyte activation in the hippocampus, along with increased permeability of the blood–brain barrier (BBB) in the hippocampus.
N. Saiyasit [18]	2020	Wistar rats	Long-term HFD showed hippocampal synaptic deplasticity and decline in cognitive functions.
Xi [19]	2019	APP transgenic mice	Administration of HFD was associated with increased amylin deposits in the hippocampus, along with brain aging and Aβ42 deposition.
G.S. Prabhu [20]	2021	Sprague Dawley rats	Long-term HFD feed resulted in significant reduction in number of surviving neural cells in hippocampus.
Guaraldi M [21]	2018	C57BL/6 mice	Treatment with HDF showed increase in ROS in hippocampus, apolipoprotein E alteration, and adverse effects on learning and memory.
Khazen, T [22]	2019	Sprague Dawley rats	Synaptic plasticity in the hippocampus was eliminated by juvenile HFD. Adult HFD enhanced in vivo LTP and object-location memory.
Lizarbe [23]	2019	C57BL/6J mice	HFD disturbed the hippocampus-dependent spatial memory with reduced levels of vesicular glutamate transporter vGlut1 and vesicular GABA transporter.
Freeman LR [24]	2012	Fischer 344 rats	HFD therapy exacerbated hippocampal microgliosis and disrupted the integrity of the blood–brain barrier.
Chou, M [25]	2022	Wistar-Kyoto rats	Long-term HFD significantly decreased the number of astrocytes and tyrosine hydroxylase-containing neurons in the substantia nigra and locus coeruleus.
Cheng, J [26]	2021	C57BL/6 mice	Reduction in APN and Nrf2 expression in the hippocampus of the HFD group.
Crescenzo, R [27]	2019	Sprague Dawley rats	HFD was observed to lower antioxidant defenses and raise the amount of PGC-1α and UCP2 in the hippocampal regions.
Nuthikattu, S [28]	2019	Mice	The hippocampus microvasculature was regulated at multiple molecular levels with prolonged ingestion of the Western diet.
Khedr, S. A [29]	2018	Wistar rats	HFD exposure showed cognitive impairment and lesser CA1 synapses, along with reduced BDNF levels.
G.S. Prabhu [30]	2021	Sprague Dawley rats	HFD supplement from young age throughout childhood, increased the TG, cholesterol and LDL levels, thus resulting in significant reduction in surviving neural cells in CA3 subregion of hippocampus.
Martinelli I [31]	2022	Wistar rats	HFD resulted in downregulation of acetylcholinesterase (AChE) enzyme, both in the frontal cortex and hippocampus.

## Data Availability

No new data were created or analyzed in this study. Data sharing is not applicable to this article.

## Abbreviations

HFD—High-fat diet, CA— Cornu Ammonis, LDL—Low Density Lipoprotein, HDL—High Density Lipoprotein, TG—Triglyceride, CCA—Common carotid artery, BMI—Body Mass Index, BDNF—Brain-derived neurotrophic factor.
